# Immune Response among Patients Exposed to Molds

**DOI:** 10.3390/ijms10125471

**Published:** 2009-12-21

**Authors:** David A. Edmondson, Christy S. Barrios, Trevor L. Brasel, David C. Straus, Viswanath P. Kurup, Jordan N. Fink

**Affiliations:** 1 The Allergy/Immunology Department at Ear, Nose and Throat Associates of North Central Wisconsin, 2801 Westhill Dr. Wausau, WI 54401, USA; E-Mail: dedmondson@westernu.edu (D.A.E.); 2 Department of Pediatrics and Medicine, Allergy and Immunology Division, Medical College of Wisconsin, 8701 Watertown Plank Road, Milwaukee, WI 53226, USA; E-Mails: vkurup1@yahoo.com (V.P.K.); jfink@mcw.edu (J.N.F.); 3 Lovelace Respiratory Research Institute, 2425 Ridgecrest Dr. SE, Albuquerque, NM 87108, USA; E-Mail: tbrasel@lrri.org (T.L.B.); 4 Department of Microbiology and Immunology, Texas Tech University Health Sciences Center, 3601 4th Street Mail stop 6591, Lubbock, TX 79430, USA; E-Mail: david.straus@ttuhsc.edu (D.C.S.); 5 Clement J. Zablocki Veterans Affairs Medical Center, Research Services 151-1, VA Medical Center, 5000 W. National Ave, Milwaukee, WI 53295, USA

**Keywords:** immune response, molds, mycotoxins, clinical symptoms

## Abstract

Macrocyclic trichothecenes, mycotoxins produced by *Stachybotrys chartarum*, have been implicated in adverse reactions in individuals exposed to mold-contaminated environments. Cellular and humoral immune responses and the presence of trichothecenes were evaluated in patients with mold-related health complaints. Patients underwent history, physical examination, skin prick/puncture tests with mold extracts, immunological evaluations and their sera were analyzed for trichothecenes. T-cell proliferation, macrocyclic trichothecenes, and mold specific IgG and IgA levels were not significantly different than controls; however 70% of the patients had positive skin tests to molds. Thus, IgE mediated or other non-immune mechanisms could be the cause of their symptoms.

## 1. Introduction

The “mold related illness” (MRI) is a controversial condition consisting of primarily non-specific symptoms such as headache, rhinorrhea, fatigue, memory loss, and eye irritation [1–6]. Although inhalation of high concentrations of mold spores has been reported to cause respiratory distress such as hypersensitivity pneumonitis (HP), allergic rhinitis and asthma exacerbations, the relationship between mold exposure and clinical outcomes remain unclear, and as a result MRI remains a contentious diagnosis [1,3]. Presently, there are three accepted pathophysiological mechanisms in mold induced disease. These mechanisms include: (a) infection by the organism, (b) generation of a deleterious immune response (e.g., allergy or HP), and c) toxic-irritant effects from mold metabolites (e.g., mycelial components, mycotoxins, or volatile organic compounds) [7–9].

Occupants of mold-contaminated environments, particularly water-damaged buildings, develop symptoms that involve multiple organs and systems including the central nervous system (CNS) and the immune system in addition to pulmonary diseases, allergy, and inflammatory reactions [10–13]. Immunological dysfunction has been proposed by those who have demonstrated immunological changes in patients after mold exposure [14]. These changes include elevations of IgG, IgM, IgA, and IgE in addition to increased CD3, CD4, CD8, CD20, and decreased CD56. An accepted practice to evaluate allergic responses is the measurement of specific IgE to molds [15,16]. Mold-specific IgG has been primarily utilized in the evaluation of those with HP or allergic bronchopulmonary aspergillosis (ABPA), although immunoprecipitation remains the standard test for these diseases [8]. IgG antibodies directed to molds can be useful indicators of exposure [17,18]. In contrast, the majority of studies performed in different countries found similar symptom profiles in correlation with contaminated buildings [19]. The objective of this study was to evaluate the signs, symptoms, serum immunologic markers including IgG, IgA, and IgE (the latter *via* skin testing), mold specific peripheral blood mononuclear cells (PBMC) proliferative responses, and serum macrocyclic trichothecene levels in individuals complaining of mold related symptoms.

## 2. Results and Discussion

A total of 33 patients were evaluated. Sixteen patients (48%) were male and 17 were female (52%). Their ages ranged from 10 to 57 years. The demographic characteristics are summarized in Table 1. The patients reported that sites of exposure were home (88%), school (3%), and work (9%). Twenty-one percent (7/33) had a previous history of asthma and 18% (6/33) had a previous history of allergic rhinitis. Seventy percent (23/33) had pets in the home and 45% (15/33) were exposed to environmental tobacco smoke. The presenting symptoms (Table 2) in order of predominance were rhinitis (82%), respiratory symptoms (73%), cough (58%), ocular pruritus (52%), gastrointestinal symptoms (48%), headache (42%), fatigue (36%), and central nervous system symptoms (30%). Thirty-seven percent of those studied had abnormal physical exam findings that included pale nasal mucosa and pharyngeal “cobblestoning”.

Seventy percent (23/33) of the patients had immediate wheal and flare skin reactivity to molds (Table 3). Thirty-three percent (11/33) were positive to mold *via* intradermal testing only. Nine percent (3/33) were positive only to environmental allergens; including tree, grass, ragweed, mites, cat and dog. Seventy-nine percent (26/33) were positive to at least one of the above mentioned antigens.

The mold surveys performed in patients’ homes reported *Chaetomyces* (18%), *Acrosporium* (15%), *Stachybotrys* (39%), *Aspergillus* (55%), *Acremonium* (6%), *Scopulariopsis* (9%), *Rhodotorula* (9%), *Cladosporium* (30%), *Aureobasidium* (6%), *Penicillium* (30%), *Epicoccum* (6%), *Phoma* (3%), and *Alternaria* (6%).

**Figure 1.**
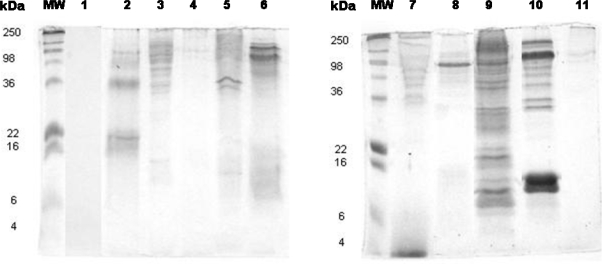
SDS-PAGE gel was loaded with 15 μg of protein per lane of *A. fumigatus* (2), *A. versicolor* (3),* A. terreus* (4), *A. flavus* (5), *Penicillium notatum* (6), *Stachybotrys chartarum* (7), *Trichoderma* (8), *Alternaria alternata* (9), *A. ochraceus* (10), and *Cladosporium herbarum* (11). Molecular weight standard as indicated by MW and growth medium (1).

The SDS-PAGE profiles of all the antigens prepared from the molds showed the presence of protein bands. Some mold extracts presented few bands (e.g., *Trichoderma*), while others presented more than ten (e.g., *Alternaria alternata* and *A. ochraceous*) (Figure 1).

**Figure 2.**
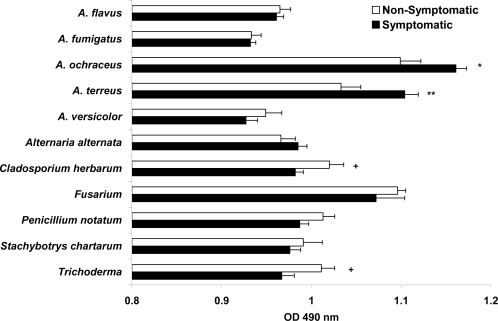
Comparison of levels of specific IgG antibodies to mold antigens, as determined using ELISA. For each sample, the same amount of coated extract was used. All sera were diluted 1:100, and all reactions were stopped simultaneously. The results are presented as mean and standard error of OD_490_ values. The */+ symbols represent statistical differences between groups, *p < 0.05, **p < 0.01 symptomatic *vs.* non-symptomatic; +*p* < 0.05 non-symptomatic *vs.* symptomatic groups.

Our study evaluated serum mold-IgG, -IgA, and -IgE antibodies (the latter *via* skin testing) as particular mold exposure markers. Thus, sera of all individuals were tested for mold specific-IgG and -IgA levels by ELISA and expressed as mean and SEM. Serum *A. ochraceus*- and *A. terreus*-IgG levels were shown to be significantly higher in symptomatic individuals compared to non-symptomatic individuals (*p* = 0.011 and *p* = 0.006 respectively). Conversely, serum *Cladosporium herbarum*- and *Trichoderma*-IgG levels were found to be significantly lower in symptomatic patients than in normal controls (*p* = 0.024 and *p* = 0.020 respectively). The remaining mold extracts-IgG levels were not significantly different between symptomatic and non-symptomatic groups (Figure 2).

**Figure 3.**
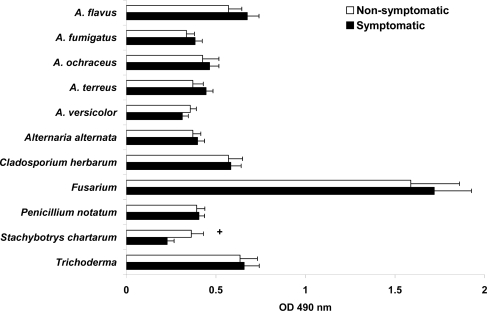
Levels of specific IgA antibodies to mold antigens are presented as OD_490_ values. Data are presented as mean ± SEM. The “*p*” value designated as +non-symptomatic *vs.* symptomatic < 0.05.

IgA levels are displayed in Figure 3. Higher serum *Fusarium-*IgA levels were present for both symptomatic and non-symptomatic populations (1.719 and 1.588 OD_490_ values respectively in diluted 1:100 serum) without reaching statistical difference among the groups. Unexpectedly, serum *Stachybotrys chartarum*-IgA levels were significantly higher (*p* < 0.049) among the non-symptomatic population than in those with mold related symptoms. The remaining mold-IgA OD_490_ values obtained with sera diluted 1:100 showed lower values and no statistical difference between groups.

**Figure 4.**
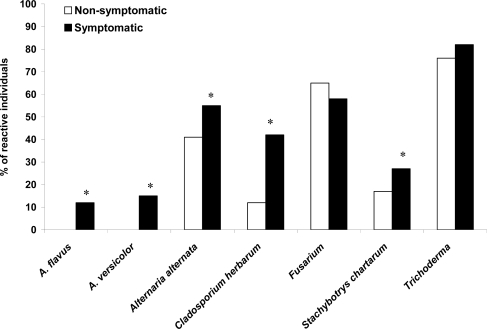
Proliferative responses of PBMC from symptomatic (filled bars) and non-symptomatic (clear bars) populations. Results are given as percent of reactive individuals to diverse mold antigens after *in vitro* stimulation. Chi-square analysis was performed to assess percent data; asterisks represent “*p*” value *<* 0.05.

Mold extract induced stimulation of PBMC was investigated in symptomatic patients with suspected mold induced illness. Figure 4 shows the degree of stimulation of PBMC to different fungal antigens by the symptomatic individuals. Patients with mold related complaints reacted to *Trichoderma* (82% *vs.* 76% of non-symptomatic individuals), *Alternaria alternata* (55% *vs.* 41%; *p* < 0.05), *Cladosporium herbarum* (42% *vs.* 12%; *p* < 0.05), *Stachybotrys chartarum* (27% *vs.* 17%; *p* < 0.05), *A. versicolor* (21% *vs.* below detectable limits; *p* < 0.05) and *A. flavus* (12% *vs.* below detectable limits; *p* < 0.05). Both non-symptomatic and symptomatic individuals specifically reacted to *Fusarium* with high prevalence (65% and 58%, respectively).

Lastly, the presence of trichothecene mycotoxin from *S. chartarum* was determined in sera from symptomatic and non-symptomatic individuals. The sera levels of trichothecene, determined by competitive ELISA in OD_450_ values and percentages of inhibition, did not show any significant difference between the symptomatic individuals compared to the non-symptomatic population (data not shown).

Although the potential of different molds or fragments to cause or aggravate adverse health effects remains unclear, the complaints associated with mold exposure continue to cause public and medical concern [6]. Mold is a greater hazard for persons with impaired host defenses or those with existing respiratory problems such as asthma and mold allergies. In recent years, a group of intermittent symptoms has been reported by individuals in connection to mold exposure [1,7,20–22].

In the present study we evaluated 33 individuals aged 10–57 years with rhinitis, cough, other respiratory symptoms, ocular pruritus, gastrointestinal symptoms, headache, fatigue, and central nervous system symptoms that were attributable to mold exposure. While abnormalities were found in the clinical examination of 37% of symptomatic individuals, the patients with mold related health complaints did not show statistical significance in the humoral immune responses. IgE-mediated responses were determined by SPT and IDT to identify the causative mold allergens in the exposed individuals. Seventy percent of the symptomatic population (Table 3) had an immediate wheal and flare reaction to molds tested by SPT and IDT.

Our group recently studied patients presenting to an allergy and asthma center with the chief complaint of “toxic mold exposure”. The symptoms included rhinitis, cough, headache, respiratory symptoms, CNS symptoms, and fatigue. The physical examination revealed pale nasal mucosa, and rhinorrhea. Fifty-three percent of the patients had skin reactions to molds. Allergic, rather than toxic, responses seemed to be the major cause of symptoms in the studied group [2]. Khalili *et al.*, described the clinical characteristics of 50 patients with complaints of illness attributed to mold exposure in the home or workplace [1]. There was no consistent set of symptoms among patients having an average of more than eight symptoms; most patients reported a family or personal history of allergy or asthma. Three quarters of the patients had abnormal physical examination results, with inflammation of the eye or skin and congestion. The authors concluded that the majority of people examined had allergic reactions, not reactions to toxins [1].

Measurements of anti-IgG -IgE and -IgA antibodies had been used to evaluate mold exposure in patients with hypersensitivity symptoms. In our study, mold-IgE measurements *via* skin testing was found to be positive in 70% of the symptomatic individuals, while only *A. ochraceous*- and *A. terreus*-IgG levels were determined significantly higher in the symptomatic population. A study showed that mold-IgG antibodies and mold exposure could not be correlated in children attending moisture mold-damaged schools, but their allergic conditions were associated with IgG antibodies to the molds [23]. Contrary to our findings, another study showed that serum IgA levels against *S. chartarum* of patients with asthma or mycotoxicosis were significantly increased compared to normal controls or asymptomatic groups, while IgG levels showed slight enhancement. IgE levels did not differ among the three groups of subjects examined [24].

In this study, although 39% of the surveyed homes reported the presence of *Stachybotrys* (informed by different evaluators), the levels of trichothecenes in sera were not significantly different from the non-symptomatic individuals. It is possible that the subjects in this study were never exposed to macrocyclic trichothecenes mycotoxins by any route or that some individuals had macrocyclic trichothecenes mycotoxins in their sera, but the level of free toxin quickly decreased below the detection limit. As Yike *et al.*, have shown, macrocyclic trichothecene mycotoxins rapidly bind to serum albumin and are quickly removed from the blood stream [25]. Brasel *et al.*, found no difference in trichothecene levels in sera of individuals with reported indoor mold exposure that was not specific for *S. chartarum.* Conversely, serum from individuals with documented indoor *Stachybotrys* exposure showed significantly higher levels of serum trichothecene [26]. Recently trichothecenes has been effectively detected in human tissue and body fluids samples, such as urine, sputum from patients exposed to mycotoxin producing molds in the environment [27].

We recognize that our study is limited by different factors. First, the lack of mold extract standardization, as molds are highly responsive toward their growth environment. We have used both commercial mold extracts and culture filtrates from formalin-killed growths prepared in our laboratory, preparations that could considerably change their reactivity potential. The potency of the used extracts may be different since the final extract is highly dependent on how the molds grow. In addition, most of the commercial extracts are made of mycelia, yet human clinical symptoms are caused by spore inhalation. And finally, we also are aware that evaluation of mold sensitivity is difficult due to cross-reactivity between diverse molds compositions and protein structures [28–30]. In this study three symptomatic individuals appeared to be atopic, i.e. had skin reactions to allergens, but only to non-mold ones and another four patients did not appear to be atopic at least by skin testing. Yet unable to establish direct association between mold related complains and immunological evaluations, we could detect positive skin test to molds in 70% of the symptomatic individuals.

## 3. Experimental Section

We evaluated 33 patients who presented to the Asthma and Allergy Center at the Medical College of Wisconsin with complaints related to mold exposure. Non-symptomatic patients were normal volunteer subjects (n = 17) that had no history of detected mold incursion in their environments nor did they have any symptoms related to atopy (asthma or rhinitis). This study was approved by the human rights review committee of the Children’s Health System, Milwaukee, WI. Written informed consent was obtained from all participants. We evaluated symptoms, medical history, medication, family history, social history, and physical examination findings. A uniform questionnaire regarding mold exposure in the home, school, or work environment was reviewed. Mold surveys performed by various environmental analyses were assessed. Allergy skin prick testing (SPT) was performed on all patients followed by intradermal testing (IDT) when appropriate. Adults were tested with 39 mold extracts in 1:20 dilutions by SPT and five IDTs composed of mixtures of eight antigens each in 1:500 dilutions of the mold allergens. Mold mixtures in IDT provide good information while avoiding performing 40 separate IDTs to detect IgE to molds. The IDTs were performed in the event of a negative SPT. Irritant reactions following IDTs were not noted. Children were tested with 8–15 selected molds *via* SPT and when feasible *via* IDTs. Additional testing included ragweed, tree, grass, mite, dog, and cat allergen sensitivity. A positive SPT was defined as a wheal diameter three millimeters greater than the negative control. Mold extracts (Greer Laboratories, Lenior, LC) were administered using the Quintip skin test device (Hollister-Stier Laboratories LLC, Spokane, WA) for SPTs and *via* 27 gauge needles using separate syringes for IDTs. The asymptomatic group (n = 17) was composed of individuals exposed only to ambient mold levels and without clinical symptoms, and their living environments had no evidence of mold amplification.

The fungal cultures were carried out using a modified method of the one previously described [31]. Briefly, *A. versicolor* (ATCC 9577), *A. fumigatus* (ATCC 42202), *A. terreus* (ATCC 1012), *A. flavus* (ATCC MYA-3631), *Stachybotrys chartarum* (ATCC 9182), *Cladosporium herbarum* (ATCC 28987), *Penicillium notatum* (ATCC 9179), *Alternaria alternata* (ATCC 6663), *A. ochraceous*, *Trichoderma spp.*, and *Fusarium spp.*, (the three former were isolated in our laboratory) spores were grown in 1:1 mixture of synthetic broth AOAC (Difco, Detroit, MI), and Czapek-Dox broth (Difco), with added glucose (1% w/v). Initially the cultures were rotated at 1,500 rpm for 24–48 hours at 37 °C. Ten milliliters of the culture were used as inocula for new stationary cultures, which were incubated for four weeks at room temperature in the same medium in one liter flasks. The culture filtrates from formalin-killed growths were passed through a filter paper (No. 1 Whatman Inc, Florham Park, NJ), and dialyzed against distilled water using 1,000 molecular weight cut off Spectra/Por Membrane (Spectrum Lab, Rancho Dominguez, CA) with three changes. A final filtration was performed for each dialyzed culture filtrate using a 0.45 micron Millipore filter. The filtrate was then freeze dried and stored (−70 °C). No attempt was made to inhibit or limit proteolysis during the preparation process. All extracts were assayed for protein by BCA protein assay kit (Pierce, Rockford, IL) according to manufacturer’s instruction. Extracts were also evaluated using sodium dodecyl sulphate (SDS) polyacrylamide gel electrophoresis (SDS-PAGE). Each mold extract was reduced in sample buffer (0.05 M Tris-HCl of pH6.8 with 1% SDS, 0.01% bromophenol blue, and 5% 2-β-mercaptoethanol), placed in a boiling water bath for five minutes, and applied at 15 μg of protein per lane on a stacking gel. Electrophoresis was carried out at constant current of 200 volts for one hour. The gels were stained with Coomassie Blue stain reagent (GelCode, Pierce, Rockford, IL).

Sera of all studied individuals were used to determine mold specific IgG and IgA antibody levels by enzyme linked immunosorbent assay (ELISA) as reported [32]. Blanks included buffer without antigen, sera were run in duplicate, and all controls were included in each assay. The blanks were subtracted and the optical density values (OD_490_) expressed as mean and standard error (SE) [32].

PBMC were isolated on Hystopaque (Sigma, St Louis, MO) by gradient centrifugation and washed twice with Hank’s balanced salt solution (Invitrogen). Cells were counted and resuspended in supplemented RPMI medium (RPMI-1640 containing glutamine, sodium pyruvate, penicillin-streptomycin, Invitrogen, Carlsbad, CA) [33]. Mold specific T cell proliferation was performed using isolated PBMC of all studied individuals. PBMC (1 × 10^5^ were added in triplicate to the wells of a sterile microtiter plate containing 50, 12.5, and 3.13 μg/mL of the culture filtrate extracts from *A. versicolor*, *A. ochraceous*, *A. fumigatus*, *A. terreus*, *A. flavus*, *Stachybotrys chartarum*, *Cladosporium herbarum*, *Penicillium notatum*, *Trichoderma*, *Alternaria alternata*, and *Fusarium*. After 24 hours, 20 μL of heat-inactivated fetal bovine serum (10% FBS, Invitrogen) were added to the wells to reduce non-specific background. The cultures were incubated for seven days at 37 °C in a CO_2_ incubator. Lymphocyte proliferation was determined by [^3^H]-thymidine incorporation during the final eight hours of culture by pulsing with one μCi of [^3^H]-thymidine (Amersham Biosciences, Piscataway, N.J., USA). Incorporated radioactivity was measured on a liquid scintillation counter (Packard Instruments Co., Meriden, CT). Specific proliferation was defined as antigen stimulated PBMC with counts per minute (cpm) at least three times higher that their corresponding non-stimulated cultures. The results were expressed as the percentage of reactive individuals [33,34].

Sera samples were analyzed for trichothecenes using a macrocyclic trichothecene-specific ELISA as described before [26,27,35,36], using the QuantiTox Kit (EnviroLogix, Portland, Maine). This kit incorporates highly specific polyclonal antibodies for macrocyclic trichothecenes immobilized on polystyrene microtiter wells. Prior to analysis, serum samples were individually aliquotted (200 μL) into sterile 1.5 mL microcentrifuge tubes followed by the addition of 600 μL of HPLC-grade acetonitrile. Samples were allowed to sit at room temperature for 15 minutes after which they were mixed thoroughly using a vortex mixer. They were then centrifuged at 14,500 rpm for three minutes to pellet the precipitated proteins. Supernatants were individually transferred into clean 1.5 mL glass vials. Each sample was evaporated to completion under a gentle stream of dry nitrogen and resuspended in 200 μL of pre-warmed sterile water. The warming aided in the resuspension of samples. From each extracted sample, 170 μL were mixed with 170 μL of horseradish-peroxidase-conjugated satratoxin G in separate 1.5 mL microcentrifuge tubes. The tubes were vortexed to ensure proper mixing. All samples and controls were added to wells in triplicate. Wells were covered and incubated at room temperature on a plate rocker for 45 minutes after which wells were washed five times with PBS using an automatic plate washer. Wells were blotted dry on clean paper towels. Immediately, 100 μL of tetramethylbenzidine substrate solution was added to each well. This was allowed to incubate at room temperature under reduced lighting for 15 minutes. To stop the reaction, 100 μL of 1N hydrochloric acid were added to each well. Wells were read at 450 nm using a microtiter plate reader. Data were expressed as mean and ± SEM of OD_450_ nm values and percentages of inhibition (PI). PI was derived from raw data and was based on patient samples compared with normal human serum control and a positive control sample spiked with Roridin A. Controls were run in parallel with test samples. The PI represents the degree of inhibition the test samples had on the capability of the satratoxin G-horseradish peroxidase (HRP) conjugate to bind to the immobilized antibody and was calculated using the following equation: PI = 100 × 1 – [(OD_450_ sample – background)/(OD_450_ control – background)].

Differences between the means of groups were analyzed with Microsoft Excel and Minitab for Windows programs. The two tails student *t*-test was used assuming unequal variances to compare levels of specific antibodies to mold antigens, while Chi-square analysis was performed to assess percent of reactive individuals to molds (proliferative responses); “*p*” values below 0.05 were considered significant.

## 4. Conclusions

The results of the present study suggest that the symptoms among mold exposed subjects, might be due to hypersensitivity to molds or mediated by some other immune or non-immune mechanisms. However, additional experimentation needs to be performed to clarify this relationship.

## Figures and Tables

**Table 1.** Demographic Characteristics of Patients Reviewed (n = 33).

**Characteristics**	**Children (n = 7)**	**Adults (n = 26)**	**Total (n = 33)**
**Gender**			
**Male**	4 (57%)	12 (46%)	16 (48%)
**Female**	3 (43%)	14 (54%)	17 (52%)

**Age, mean (range), y**	13 (10–17)	41 (18–57)	35 (10–57)

**Past medical history**			
**Asthma**	0 (0%)	7 (27%)	7 (21%)
Allergic rhinitis*	1 (14%)	5 (19%)	6 (18%)

Mold exposure site**			
**Home**	7 (100%)	22 (85%)	29 (88%)
**School**	0 (0%)	1 (4%)	1 (3%)
**Work/Office**	0 (0%)	3 (11%)	3 (9%)

**Social history**			
Smoke exposure***	3 (43%)	12 (46%)	15 (45%)
Pets exposure****	17 (65%)	6 (86%)	23 (70%)

* A previous physician diagnosed allergic rhinitis and this was reported at our initial visit.

** Location where exposure took place, data originated from the uniform questionnaire reported by patients.

*** Patients are smokers or there is second hand smoke exposure.

**** Patients own pets and are exposed on a regular basis.

**Table 2.** Common Presenting Symptoms of Patients with Mold Exposure.

**Symptoms**	**Children (n = 7)**	**Adults (n = 26)**	**Total (n = 33)**
**Rhinitis**	7 (100%)	20 (77%)	27 (82%)
**Cough**	5 (71%)	14 (54%)	19 (58%)
**Headache**	3 (43%)	11 (42%)	14 (42%)
Respiratory symptoms*	4 (57%)	20 (77%)	24 (73%)
**Ocular pruritus**	5 (71%)	12 (46%)	17 (52%)
CNS symptoms**	0 (0%)	10 (38%)	10 (30%)
**Fatigue**	0 (0%)	12 (46%)	12 (36%)
Gastrointestinal symptoms***	3 (43%)	13 (50%)	16 (48%)
**Epistaxis**	0 (0%)	1 (4%)	1 (3%)
**Dysuria**	0 (0%)	4 (15%)	4 (12%)

* Symptoms include dyspnea, wheeze, chest tightness, and shortness of breath.

** Symptoms include dizziness, anxiety, weakness, memory loss, and shaking.

*** Symptoms include nausea, vomiting, and abdominal pain.

**Table 3.** Mold Panel for Skin Prick Test (SPT) and Intradermal Testing (ITD)*.

Mold Antigens for IDT**		Mold Antigens for SPT**	

**Mold Group I**	13 (39%)	*Alternaria*	8(24%)
		*Aspergillus fumigatus*	4(12%)
		*Aspergillus niger*	0 (0%)
		*Botrytis cinerea*	0 (0%)
		*Candida albicans*	0 (0%)
		*Candida tropicalis*	0 (0%)
		*Cephalosporium*	0 (0%)
		*Cephalothecium roseum*	0 (0%)

**Mold Group II**	16 (48%)	*Chaetomium globosum*	0 (0%)
		*Cladosporium fulvum*	0 (0%)
		*Curvularia spicifera*	2 (6%)
		*Epicoccum nigrum*	2 (6%)
		*Fusarium oxysporum*	2 (6%)
		*Geotrichum candidum*	0 (0%)
		*Cliocladium fimbriatum*	0 (0%)
		*Helminthosporium*	1 (3%)

**Mold Group III**	16 (48%)	*Hormodendrum*	4 (12%)
		*Microsporum audouinii*	0 (0%)
		*Microporum canis*	0 (0%)
		*Mucor racemosus*	0 (0%)
		*Neurospora intermedia*	1 (3%)
		*Nigrospora oryzae*	2 (6%)
		*Paecilomyces variotii*	1 (3%)
		*Papularia montagnei*	0 (0%)

**Mold Group IV**	11 (33%)	*Penicillium notatum*	2 (6%)
		*Phoma destructive*	1 (3%)
		*Pullularia pullulans*	1 (3%)
		*Rhizopus stolonifer*	1 (3%)
		*Rhodotorula rubra*	1 (3%)
		*Saccharomyces*	0 (0%)
		*Scopulariopsis*	0 (0%)
		*Spondylocladium*	1 (3%)

**Mold Group V**	6 (18%)	*Sporotrichum*	1 (3%)
		*Stachybotrys chartarum*	1 (3%)
		*Stemphylium herbarum*	2 (6%)
		*Phycomyces*	0 (0%)
		*Syncephalastrum*	0 (0%)
		*Tetracoccosporium*	0 (0%)
		*Trichoderma rufa*	1 (3%)
		*Trichophyton rubrum*	1 (3%)
**Total**	**19 (58%)**		**13 (39%)**

**Controls**		Negative Control	0 (0%)
		Histamine	33 (100%)

* Grouped antigens (eight per group) for intradermal testing (IDT) were divided alphabetically as provided by the extract manufacturer. 23 (70%) of the total symptomatic population was IPT and/or IDT positive.

** Individuals from the symptomatic group were frequently positive for more than one mold antigen and mold group.
